# Book Reviews

**Published:** 1893-06

**Authors:** 


### Book Reviews.

A System of Genito-Urinary Diseases, Syphilology, and Dermatology, by various authors. Edited by Prince A. Morrow, A. M., M. D., Clinical Professor of Genito-Urinary Diseases; formerly Lecturer on Dermatology in the University of the City of New York; Surgeon to the Charity Hospital, etc. With illustrations. In three large 12mo volumes. Vol. I., Genito-urinary Diseases. Sold only by subscription.

The most comprehensive system of the diseases named in this title that has ever been published is contemplated by the editor, of which the present is the first volume. Two others are to follow, making the work complete in three volumes. It is encyclopedic in character, and the contributors to Vol. I. number thirty-two authors and writers distinguished in the several departments on which they discourse.

The Anatomy and Physiology of Genito-urinary Organs are considered in the first section of the book by George Woolsey, M. D. The next subject, Diseases of the Penis, is written upon by Raymond Guiteras, M. D. This will be found a most interesting section, though it is comparatively brief. Diseases and Injuries of the Urethra form the subject of the next section, and are discoursed upon by F. Tilden Brown, M. D. Various operations for hypospadias and epispadias are described and elaborately illustrated. The Etiology of Urethritis is next considered by S. Lustgarten, M. D.

Acute Urethritis, or Gonorrhea, is elaborately handled by George Emerson Brewer, M. D. This chapter is one of great satisfaction, and is full of valuable thought with reference to all phases of this most serious malady. It is a great mistake for physicians to regard this disease as of little importance and to treat it in a flippant or careless manner. There is more danger lurking in a neglected or uncured gonorrhea than in any other venereal disease. Everyone should be familiar with what Dr. Brewer has written.

The next section is upon Chronic Gonorrhea, or Gleet, and is briefly dealt with by William K. Otis, M. D. Endoscopy, by M. Clotz, M. D., is a most interesting chapter. It is important that one should be expert in order to gain such information from the endoscope as is required to make it useful in the diagnosis of disease. The author has fully set forth the details of the manipulation of this instrument. Gonorrheal Ophthalmia, by James A. Andrews, forms the subject of the next section of the book, which is most important in its bearings. Every obstetrician should know how to treat this direful malady, and especially should he know how to prevent it. Gonorrheal Rheumatism is dealt with by Frank Hartley, M. D., and Gonorrhea of the Rectum, Mouth, Nose, Ear, Umbilicus, and Axillary, by James D. Tuttle, M. D. Stricture of the Urethra is elaborately dealt with by J. William White, M. D., in the next seventy pages of the book. This is one of the most important chapters for the genito-urinary surgeon, and one that will command the attention of teachers and specialists throughout the land.

Diseases of the Prostate are treated of in the next section by Dr. W. T. Belfield. This important organ has been much neglected by general practitioners, and should be studied in the light of this recent literature. Dr. Joseph D. Bryant writes on Functional Disorders of Micturition, and Dr. Eugene Fuller discourses on the Diagnostic Significance of Pathological Modification in the Urine. Next follows a chapter on Urinary Fever, by Dr. J. A. Fordyce, and then Dr. Willy Meyer discourses upon Cystoscopy. This is one of the most interesting chapters in the book, and is written by a masterful hand; it is well illustrated and deserves careful study.

The Cystites, by Dr. Samuel Alexander, forms the subject of the next section, and this is followed by Dr. George Ryerson Fowler on Diseases and Injuries of the Bladder. Dr. Fowler is a surgeon of experience, and his section is ably discussed. Tumors of the Bladder are treated of by Dr. Francis Sedgwick Watson, and his plates illustrating carcinoma are most interesting. Suprapubic Cystotomy is described in detail in this chapter, and then Dr. Arthur T. Cabot writes a section on Stone in the Bladder. This is illustrated with numerous wood-cuts and some reproduced photographs illustrating Litholapaxy and Lithotomy. This is another of the essential chapters of the book and should be carefully studied to be appreciated.

Surgical Diseases of the Kidney are handled by Dr. Lewis A. Stimson in his usual masterly style. In view of the recent advances in the treatment of these diseases, such a contribution as this is a useful addition to the literature on the subject. Tuberculosis Uro-genitalis, by Dr. John P. Bryson, is next considered, and then Diseases of the Scrotum, by Dr. Charles W. Allen; then follow chapters on Diseases of the Testicle, by Dr. Edwin C. Burnett, Dr. James Bell, and Dr. John P. Bryson; next, Hydrocele and Spermatocele are dealt with by Dr. John A. Wyeth and Dr. W. W. Van Arsdale. Varicocele is treated upon by Dr. Edward L. Keyes, who is an acknowledged master in genito-urinary surgery. Diseases of Seminal Vesicles, by Dr. Paul Thorndike, form the subject of the next section, followed by a chapter on Functional Disorders of the Male Sexual Organs, by Dr. Prince A. Morrow; this includes Spermatorrhea, Impotence, and Sterility. Dr. Andrew F. Currier treats of Gonorrhea in the Female and its Complications, which forms the final chapter in the book. A copious index is furnished at the end of the volume.

Taken altogether, this is the most satisfactory exposition of the diseases in the genito-urinary system which has been put forth, and when the other volumes are issued it will constitute an encyclopedia upon the subject which must necessarily be found on the book-shelves of every teacher and specialist.

---

A TEXT-BOOK OF PRACTICAL THERAPEUTICS, with especial reference to the application of remedial measures to disease and their employment upon a rational basis. By HOBART AMORY HARE, M. D., B. Sc., Professor of Therapeutics and Materia Medica in the Jefferson Medical College of Philadelphia; Laureate of the Royal Academy of Medicine in Belgium, of the Medical Society of London, etc.; President of the Section of Therapeutics in the Pan-American Medical Congress. Third edition. Enlarged and thoroughly revised. Cloth, $3.75; leather, $4.75. Philadelphia: Lea Brothers & Co. 1892.

The first edition of this book was published in September, 1890, and the ink was scarcely dry from the reviewer's pen before a second edition was demanded, which was put forth in May, 1891. In less than two years a third edition becomes necessary, and these facts alone would seem to indicate both the popularity and usefulness of this work. It will be remembered that Hare's System of Therapeutics, an encyclopedia work on a unique plan and a comprehensive scale, has been published only very lately. Taking these facts altogether, it would appear that the author is not only a master of the subject, but a man of marvelous industry, who finds time amidst a busy practice and activity in teaching to pursue to a finish such a vast amount of literary work, in such a comparatively short time.

This volume is properly named as a Text-book of Practical Therapeutics, which is emphatically the case from book-cover to book-cover. In this third edition will be found all the thoroughness of former editions, together with the latest and most reliable results obtained in the treatment of disease by those who have employed the newer remedies. Hare says in his preface:

> In this edition, directions are given for the use of drugs of such recent introduction as salophen, guaiacol diuretin, europhen, thiol, phenocoll hydrochloride, piperazine, dermatol, pental, terpine hydrate, terpinol, and the salts of stontium. Much information has also been added to the articles on such drugs as salol, phenacetine, menthol, and similar comparatively recent remedies. Another important addition is a list of drugs arranged as far as possible according to their physiological action, and a list of definitions of the terms used to designate classes of drugs.

The student and practitioner will not be disappointed in this statement, because its truthfulness is verified over and over in the text of the book. The volume contains a valuable table of doses of remedies, a table of relative weights and measures in the metric and apothecaries' system, besides an index of drugs and remedial measures, together with an index of diseases and remedies. It is hardly possible for student, teacher, or practitioner to do without this work.

---

Hand-Book of Materia Medica, Pharmacy, and Therapeutics, including the Physiological Action of Drugs, the Special Therapeutics of Disease, Official and Practical Pharmacy, and Minute Direction for Prescription Writing. By Samuel O. L. Potter, A. M., M. D., M. R. C. P., Lond., Professor of the Theory and Practice of Medicine in the Cooper Medical College, of San Francisco; Visiting Physician to St. Luke's Hospital; Author of Quiz Compends of Anatomy and Materia Medica, An Index of Comparative Therapeutics, and A Study of Speech and its Defects. Formerly A. A. Surgeon, U. S. Army, and Brigade-Surgeon, N. G. of California. Fourth edition, revised. Octavo volume, pp. xii.—781. Price, $4.00. Philadelphia: P. Blakiston, Son & Co., 1012 Walnut street. 1893.

We have noticed previous editions of this work in our columns, and can only say that this new edition is a continuation of the good form of the previous volumes. It has been accepted as a text-book by the colleges, and is found in a large number of libraries. The painstaking work of the author is shown by the fact that he has grouped together the salient features of the subject in a very accessible form, which is neither too small nor too voluminous. He lays great stress upon the importance of the knowledge of incompatibles, and every student should familiarize himself with this subject before trusting himself to write prescriptions. The arrangement of the volume is one of the best we have seen, and the thumb-index makes it of easy reference. We presume that teachers, students, as well as practitioners, will avail themselves of the opportunity to purchase for the sum of $4 one of the best standard works on Materia-Medica, Pharmacy, and Therapeutics, published in the English language.

---

Transactions of the American Surgical Association. Volume the tenth. Edited by J. Ewing Mears, M. D., Recorder of the Association. Philadelphia: Printed for the Association and for sale, by Wm. J. Dornan. 1892.

The tenth annual volume of this distinguished association is abreast with the former issues both, in scientific interest and admirable make-up. The fact that the association will meet in Buffalo almost simultaneously with the appearance of the JOURNAL, and under the presidency of that distinguished surgeon, Dr. Nicholas Senn, lends an added interest to the proceedings of the society this year, which, no doubt, will be watched with profit by the profession residing in Western New York.

In the present volume, Dr. N. P. Dandridge, of Cincinnati, contributes a most interesting paper on the Surgery of the Tongue, which was amply discussed. This forms a valuable addition to the literature of this important department of surgery. Another paper which will attract attention, and be quoted as authority is entitled Treatment of Fractures of the Lower End of the Humerus and Base of the Radius, by Dr. John B. Roberts, of Philadelphia.

We commend the entire volume to the careful reading of surgeons, and all who make any pretense to the practice of surgery.

Dr. J. Ewing Mears, the accomplished Secretary, has issued a brochure, which may be termed an appendix in separate form to Volume X., containing a complete list of the officers and fellows of the American Surgical Association from the date of its organization in 1880 to 1892, together with an alphabetically arranged index of the ten volumes which the association has issued. This is a valuable supplement to the work of the association for a decade.

---

A MANUAL OF MEDICAL JURISPRUDENCE. By ALFRED SWAINE TAYLOR, M. D., F. R. S. Revised and edited by Thomas Stevenson, M. D., London, Fellow of the Royal College of Physicians, London; Lecturer on Medical Jurisprudence and Chemistry at Guy's Hospital; Examiner in Forensic Medicine in the University of London; External Examiner in Forensic Medicine in the Victoria University; Official Analyst to the Home Office. Eleventh American, edited with citations and additions from the twelfth English, edition. By Clark Bell, Esq., President of the American International Medico-Legal Congress of 1893. Honorary Member of the Medico-Legal Society of France; of the Society of Mental Medicine of Belgium; Ex-President of the Medico-Legal Society of New York, etc., etc. *Quod hodie exemplis tuemur mox inter exempla erit.* Philadelphia: Lea Brothers & Co. 1892.

Taylor's Medical Jurisprudence has been so long before the American profession, where it has been considered a guide to the subject of Forensic Medicine and a text-book in the colleges, that little remains to be said after we have noted the fact that the eleventh American edition has been revised and brought forward to include the immediate present. This work has been performed by an eminent member of the New York bar, the Honorable Clark Bell, who is a writer and teacher on medical law. This edition has been made to include the twelfth English edition, and so has the very latest references. No library is complete without Taylor's Medical Jurisprudence, as its authority is accepted and unquestioned by the courts.

---

### BOOKS RECEIVED.

Cholera: Its Protean Aspects and its Management. By Dr. G. Archie Stockwell, F. Z. S., (Member New Sydenham Society, London.) In two volumes—Vols. I. and II. "Respice, aspice, prospice." Physicians' Leisure Library. Price, 25 cents: Detroit, Mich. George S. Davis. 1893.

Manual of Chemistry. A Guide to Lectures and Laboratory Work for Beginners in Chemistry. A Text-book specially adapted for Students of Pharmacy and Medicine. By W. Simon, Ph. D., M. D., Professor of Chemistry and Toxicology in the College of Physicians and Surgeons, Baltimore; and Professor of Chemistry in the Maryland College of Pharmacy. New (fourth) edition. In one 8vo volume of 490 pages, with forty-four wood-cuts and seven colored plates, illustrating fifty-six of the most important chemical tests. Cloth, $3.25. Philadelphia: Lea Brothers & Co. 1893.

Medical Pocket Atlas. Obstetrics. Part I. Labor delineated in ninety-eight plates. By O. Schaeffer, M. D., Assistant at the University Frauen-Klinik, in Munich. Translated and published under the supervision of J. Clifton Edgar, M. D., Adjunct-professor of Obstetrics in the University of the City of New York; Attending Physician to the New York Lying-in Hospital; Assistant Obstetric Surgeon to the New York Maternity and to the Emergency Lying-in Hospitals. Formerly Volunteer Interne in the University Frauen-Klinik, of Munich. New York: L. Hydel, Publisher, 212 E. 50th street. 1893.

A Treatise on the Theory and Practice of Medicine. By American Teachers. Edited by William Pepper, M. D., LL. D., Provost and Professor of the Theory and Practice of Medicine and of Clinical Medicine in the University of Pennsylvania. For sale by subscription only. In two volumes,—pp. 1,000. Price per volume, cloth, $5.00; sheep, $6.00; half-Russia, $7.00. Philadelphia, Pa.: W. B. Saunders, Publisher, 913 Walnut street. 1893.

Psychopathia Sexualis, with Especial Reference to Contrary Sexual Instinct. A Medico-Legal Study. By Dr. R. von Krafft-Ebing, Professor of Psychiatry and Neurology, University of Vienna. Authorized translation of the seventh, enlarged and revised, German edition. By Charles Gilbert Chaddock, M. D., Professor of Nervous and Mental Diseases, Marion-Sims College of Medicine, St. Louis; Fellow of the Chicago Academy of Medicine; Corresponding Member of the Detroit Academy of Medicine; Associate Member of the American Medico-Psychological Association, etc. In one royal octavo volume, 436 pages. Extra cloth, $3.00, net; sheep, $4.00, net. Sold only by subscription. Philadelphia: The F. A. Davis Co., Publishers, 1914 and 1916 Cherry street.

